# Valedictory Address to the Graduating Class of the Baltimore College of Dental Surgery

**Published:** 1869-04

**Authors:** E. Lloyd Howard

**Affiliations:** Professor of Anatomy.


AMERICAN JOURNAL

OF

DENTAL SCIENCE.

Vol. 11. THIRD SERIES--APRIL, 1869. No. 12.

### ARTICLE I.

### *Valedictory Address, to the Graduating Class of the Baltimore College of Dental Surgery, March 3rd* 1869.

By E. Lloyd Howard, M. D., Professor of Anatomy.

Gentlemen of the Graduating Class:—Having completed the course of studies which has been marked out for you, and after careful examination having been found well qualified to enter upon the practical duties of your profession, you are this evening admitted, as proper members, of that special branch of the great science and art of Surgery, which you have selected as the business of your lives.

In the popular understanding, you are supposed this evening, to terminate your student life; and that now, closing your books, you shall devote yourselves henceforth, in putting to practical uses the knowledge you have obtained.

But I trust there is not one amongst you, who will be content, ever to give up student life, and settle down into the mere routine practitioner. I trust there is not one here, so dead to enthusiasm, so destitute of ambition, as to be satisfied with his present acquirements, and whose only desire is to make money by their practical application! If so, then let me tell you plainly, the sooner you leave the noble field of labor into which you seek unworthily to enter, the better for all concerned!

No! Gentlemen! If you would practice your profession with any credit to yourselves, and with justice to it, you never can cease being students! The diploma which you receive this evening, should not be regarded, only as a reward for work done, but, rather as a token that you are worthy to be admitted to a broader field of labor, in the cultivation of which, you can gain the highest rewards the world offers to genius and talent!

It may seem to you inappropriate, that to night, when you justly feel yourselves entitled to at least a little respite after your arduous labors of the winter, I should come before you to preach more work, rather than take relaxation with you amid the flowers of rhetoric, and complimentary speeches. Yet I cannot but feel that the occasion has a double significance; that not only to-night do you end a long day's work; but that on the morrow you will be called again early to toil; and if I can point out to you how you may greatly lighten the burden; how you can convert the curse of labor into a blessing; if I can inspire you with courage to "go forth to meet the shadowy future with bold and manly hearts," surely this is well and not inapproprite!

Your profession, as a legitimate and honorable specialty of the Science of Medicine, has intimate relations with all the physical sciences; and to be master of your art, you must have some acquaintance with them all. With this illimitable field before you, at what point shall your studies begin; in what direction tend; where be limited? These are questions upon the proper answer to which, in great part, will depend the success of your efforts, and, principally to their solution, I propose to ask your attention this evening. And I do so, because I am profoundly impressed with the belief, that their importance is generally not sufficiently estimated, and that to a disregard of them, is due a much greater share of life failures, than is commonly supposed.

What becomes of all those remarkable young men annually sent out by the colleges—whose genius is to rule the destinies of the country; to illuminate the sciences; to regenerate society— those first men of their class? Standing a head and shoulders above their fellows in the beginning of life's battle, where are they a little later in the fight? We look for them in vain; others rated greatly below them in college days, now overtop them, and are far in the advance! There are certaiuly exceptions, but is it not generally true that the first men of the schools, do not retain their supremacy? Why is this? Is it from a positive degeneration of the one class; or a greater relative improvement of the other?

Undoubtedly one great cause, is to be found in the fact, that men attain their intellectual developement at very varying ages; while one reaches his full growth at eighteen, another does not gain it at twenty-five. The best men of the colleges, are commonly those, who are only precocious; and it is natural to expect, that a little later in life, they will be overtaken by many, who from a slower development,—not any essential inferiority,—were once below them.

But there is another cause; and it is to this that I wish more particularly to allude. The young man is often ruined by his very successes. After graduating at the head of his class; filled with a high and generous enthusiasm, he goes boldly into the world to meet life's duties! The vigor of youth and strength elate him into the vainest dreams! He fancies that he can become universal in his genius! He hopes to attain some grand elevation, whence he can look down upon, and grasp all the varied fields of knowledge! He will solve that problem that has baffled all before him, and must baffle all to come after! He will reach that pinnacle of wisdom, whence he can stretch forth his hand, and gather from every store of knowledge, with scarcely an effort! Vain dreamer,—aiming at that, which is the sole prerogative of Deity!

Is this a fancy sketch? Analyze your own hearts! In some more strongly felt than in others; in many a mere indefinite, uneasy sensation; I believe there never yet lived a man of any ambition, who has not at one time experienced it!

Let one of these enthusiasts enter a large library; as he looks around upon the accumulations of bygone ages—nay, if he only take the acquirements of the present day, how baffled and impotent does he feel, in attempting to store and classify in his mind, the treasures he fain would appropriate! Heart-sick and wearied with the fruitless effort, one of two things then befall him. He either gives up in despair; declares the uselessness of the attempt; and merely lives to meet the daily requirements of life, retaining a respectable position in society, and nothing more; or else, he gropes his way along, day after day accumulating, until old age overtakes him, and finds him as unready and unsatisfied as when he began his labors; a pedant; a bookworm,—harmless, may be ornamental, but utterly useless to all the practical duties of life! Look around, you may see scores of such wrecks scattered along the highway of life!

It is a painful lesson to learn, but one that must be experienced some day, by all who would make true intellectual progress, that the mental capacity of the best of us, is limited to but comparatively narrow bounds, and the sooner we recognize the fact, and learn to fairly appreciate our abilities the better for us. Just as there is a limit to our physical force, so with the mental; and as we carefully calculate the capacity of the one, before entering upon any arduous task; so should we weigh the ability of the other, before putting it to labors that may be too great for it. He who wastes his energies in trying to attain an ideal intellectual supremacy, may be just as insane, as the inmate of the hospital, who spends his days in trying to leap to the moon; and his efforts just as barren of results.

I am led to these remarks, by the firm conviction, that the current doctrines upon intellectual cultivation, are based upon false notions of mental physiology. I believe them to be not only unsound in theory, but most disastrous in their practical applications. We are commonly taught, that the mind is a something capable of unlimited expansion; and that, consequently, in our efforts at its cultivation, we cannot fix too high a standard, or aim too loftily. As well tell the gymnast, that in his efforts at physical developement, the best exercise he can take, is to employ himself in vain attempts to leap over a mountain; as well tell the boy, that the best means he can adopt to perfect himself as a marksman, is to take the stars as his targets!

I believe this false teaching in great part responsible for the vain aspirations of young men; encouraging them to extravagant dreams, which must shortly be dissipated, leaving them often soured and disappointed, for life!

Do not think gentlemen, that I would discourage one earnest worker! Far be it from me, to check one impulse of enthusiasm! All I would do, is to point it in the proper channel; to show how that enthusiasm should be best applied; and to warn you from wasting your energies in efforts that can only lead to failure and disappointment. And let not this infirmity of our nature too greatly discourage you; within the scope of our powers is ample room for the full exercise of all our faculties, if we would but use them rightly.

I know of no more dangerous snares and pitfalls for young men, than the words "genius" and "talent." It cannot be reckoned how many are ruined by fancying themselves possessed of a sort of magic power, called "genius," which lifts them above the common herd, and is to bring them to fame and honor, by its mysterious workings, with scarcely an effort upon their part! And it seems as if those of real ability, are the ones most liable to fall into such an error! Who has not seen hundreds of such, Micawber like, "waiting for something to turn up," which means, they are waiting for the world to discover and appreciate their talents! as if, for sooth, the world had nothing better to do, than busy itself in searching out such lights under bushels! Believe me, the highest talent, if judged by its fruits, is the talent for work; the highest genius, the ability to properly choose, and direct work to definite accomplishments! Recognising then the illimitable field of study that is before you: the limited capacities of the human mind; and the fact that, "man who is born of woman, hath but a short time to live," how should your studies be directed—what knowledge is of most worth? The best general advice I can give, is that you should devote your studies chiefly to those brances of science on which your profession is most directly founded. This study should be thorough; no one point should be left untouched; all the details should be most carefully worked up. This should be your art study, on which all your best energies should be bestowed; to which all your best talents should be consecrated!

But this will not suffice; you want to be something more than mere workers in your art. It is necessary to have some information about the arts and sciences generally; about general literature; and it is in the manner of acquiring this general cultivation, that our efforts are commonly unorganized, and misdirected. Here you must not strive for too close a knowledge. Life is too short, even did our capacity admit, to accumulate the various details of all the sciences. Here you must learn to generalize; aim at grasping only the great leading principles, and do not attempt to burden your memories with a mass of facts, that can have no practical use, or significance for you. Take the science of Astronomy, for instance, it will be both interesting and useful to inform yourselves of its leading principles, and broad general facts; but to enter into its minutiæ; to learn its details, would be to you, more than a waste of time. And this is the reason the sciences are not more popular studies; not that they are uninteresting subjects in themselves, but that they become so, from the manner in which they are commonly taught. Viewed simply as a collection of facts, they are calculated to disgust the most ardent student; taken as broad generalizations, nothing can be more attractive;—full of truth, of beauty, and of poetry; they have facts, stranger than the strangest fiction, more beautiful than the highest creations of art, and the harmony of the stars is poetry itself!

Do not try then to remember too closely facts, except such as have a practical bearing for you; but rather endeavor to fill your minds with liberal, and comprehensive views.

But such advice is calculated to make you mere superficial readers and thinkers! In general matters, yes; as long as the intellectual range is so narrow, and life so short, it needs must be so. It is better in these things, to sacrifice depth for breadth, since we cannot compass both. No closeness of research can be too close and deep, in the special studies of your profession; but closeness of research, tends to mental myopia; and this tendency, can only be counteracted by cultivating enlarged views upon other subjects; so the proper balance will be preserved; and you can excel in your profession, and at the same time, be something more than the mere professional man. Let all your studies then, be intelligently directed: and do not fall into the popular error, that all knowledge is good, and that study simply consists in the accumulation of facts!

Such a course involves work; but it is a work that brings ample rewards; and which ennobles, and dignifies the worker. It is that higher form of work, that is as natural to man in his full developement, as play is to the child,—and the fool! He who would stand all the day idle, has not arrived at the perfection of manhood; he cannot know one of the greatest of life's blessings!

You have probably found difficulty in reconciling the teachings of those, who hold that work is the great curse sent upon humanity; and of the later school of philosophers and poets, who would have us believe, that work is always, and in itself, a great blessing; who preach—and the world is just now resounding with their doctrine—the "nobility of labor." The truth is to be found here, as elsewhere, I apprehend, between the extremes. At times there will come to all of us, distasteful work, that I defy all the moral philosophers of the world, to prove other than an unmitigated curse! On the other hand, artistic work, is one of the highest pleasures of life! It calls into healthy exercise all our noblest faculties! The artist, giving expression to his highest conceptions of truth and beauty, finds his greatest happiness in the work! In the higher forms of labor, the cure is abrogated! It is only where the sculptor is taken from his artistic work, and put to "hewing off the rough," that the curse is felt.

Unfortunately, the necesities of life often compel us to this work that is beneath us,—the drudgery of labor. But there is some discretionary power with us; to some extent we can select what from of work, we will put our hands to do; and when this is so, we should choose that field which demands the fullest expression of the highest faculties that are in us. For do not think the nobility of labor only attainable by the painter and the sculptor; it is open to all! There is no mind so feeble; there is no occupation so low, that cannot reach it! In all professions; in all business; in all the lower forms of labor, the mechanic and the professional man, each, in his own sphere, can attain it! The nobility of work, is in its perfectness. One mind differs from another as the stars differ in glory, but let each shine forth to its full capacity, and it is fulfiling the design of its Creator, the law of its nature. And if you will select the highest form of work that your talents can compass, and follow the golden rule to do whatsoever you put your hand to do; with all your heart, to the full perfection of your strength, there will arise such gratification in doing, such contentment in having done, that there will be no room for the curse!

You stand this evening, gentlemen, at the threshold of a noble profession! The choice is now before you, whether your work shall be a heavy burden, a mere drudgery, only to be accomplished for the sake of pecuniary renumeration; or whether, guided by study and thought, illuminated by your brother workers, it shall be to you an art-work; not only supplying the material necessities of life, but being a pleasure in itself, gaining for you credit and reputation, and it may be, fame!

If you rightly apprehend my meaning, you will perceive that I am no advocate for the current philosophy in its extreme tendencies, that work is essentially a blessing; that the noblest thing man can do, is to labor unceasingly—no matter at what, so he labor. And on the other hand, I cannot regard work, in the light of a curse, and as an essential evil. From our physical structure, and from the requirements of society we must all do some form of work. None of us are exempt from the cares of life, and to meet those cares, often imposes disagreeable tasks. I cannot give you a recipe which will be a grand specific for all the troubles and toils of life's journey; many rugged places must be encountered, and passed over. But I do hold, that by a properly systematized work, the journey can be made through an interesting and picturesque country, instead of through barren wastes; that the traveller may be stimulated to overcome many obstacles, that else might prove impassable!

Is this philosophy a mere theory, inapplicable to every day life? Not so! If you will look a little more closely at the world, and its different classes of workers, you can see it exemplified all around you! On the one hand the routinist, who feels no interest in his work, except for the remuneration it will bring him in material good. On the other, the scientific worker, who reaps the same rewards as the former,—and in greater abundance,—and in addition, feels some pleasure in his labor; gaining breadth of thought, and an increased scope to his mental powers; with the applause of his fellow workers!

You have doubtless often heard it said of general medicine—and it equally applies to your specialty—that the *study* is facinating, but the *practice* of it, the veriest drudgery. Believe it not! To the true practitioner, the study is never ended; and the longer he lives, the more interesting, does his rational practice become! It is only when the rational practice is given up, either from indolence, or from too much work, and a routine system takes its place, that there is drudgery! And here is one of the great imperfections in the workings of our professions; the young man becomes discouraged and indolent from too long waiting for practice; while the older becomes a routinist, from too much work accumulating! This can never be remedied until skill and experience demand a higher price than inexperience. If it were generally understood, that the older and more experienced the practitioner becomes, the smaller his number of cases, and the greater his charges; I apprehend his work would be more perfect, and great advantages would accrue, both to the profession, and the public.

To practice artistically then, you should regard each case that comes before you, as a special study, and believe me, if rightly viewed. you will find something of interest in every one!

But in thus devoting yourselves to your profession, avoid being too exclusive in your devotion. Life has other duties; society other claims. Yours is a liberal art; and not a mere mechanical one. The tendency of professional life is too much toward exclusiveness; and while making yourselves as perfect as may be in Dentistry, do not cease striving to gain broad, and liberal ideas upon general subjects, by a well directed study and social intercourse! As I have shown, the two need not conflict, but should be in harmony.

Indeed it is less excusable in you to neglect the cultivation of general science and literature, than in the practitioner of medicine; for your opportunities, and leisure are greater. The hours of work are never over for the physician; his leisure never certain. In dentistry you have fixed times for work, and for rest. And the rest should be passed not in unrefreshing indolence, but in the expansion and enjoyment of refined tastes and pleasures; in this way counteracting the narrowing tendencies of professional duties; obtaining true refreshment; and preventing the weariness of monotony, which an exclusive occupation will bring!

And you should remember, gentlemen, that you have assumed responsibilities, in entering this profession; responsibilities, to the profession, and to your brother workers! It now behooves you to guard jealously the honor of your calling, and see that through you, no reproach shall rest upon it! American dentistry is taking high rank as a specialty of surgery, and it is incumbent upon each of you, to aid in keeping it in its high position, and giving it additional reputation.

And now, it only remains to say farewell! Having been so long fellow-students, and workers; it is not unnatural that certain bonds of sympathy between the Faculty and yourselves, should have been formed, which may not be broken, without regret!

And as if to warn us of the temporary nature of these human relations, within the past few weeks, the ties which united us with one of our number, have been rudely sundered by the hand of death—he whose place I unworthily fill this evening! Would he had been spared to speak to you to-night the words of advice and encouragement, he was so well qualified to give! Possessed of a vigorous intellect, gifted above most men in the universality of his acquirements, while second to none in his special department of science, we shall never cease to feel his loss! A genial, courteous gentleman; a true and faithful friend; an enthusiastic worker; let us try to imitate his truth and steadfastness, as we keep in admiration his high attainments!

And yet gentlemen, the occasion cannot be a sad one to you! Having fairly earned a position in the foremost ranks of your profession, a clear field is before you!

I have felt constrained this evening to impress upon you the necessity of tempering enthusiasm with reason, and to dissuade you from indulging in extravagant dreams, that can never be realized. But on the other hand; I trust I have not obscured the fact, that within certain limits, the future can be to you, what you choose to make it; and within these limits are contained rewards and honors, sufficient to satisfy any reasonable ambition. And I should be false to myself, if I uttered one word, to discourage you from attempting them! I have too much faith in the living present; too much hope in thefuture, to permit me unnecessarily to check the ardor of a fellow-worker! I wonld have you fairly estimate your powers, clearly define the goal you may set for yourselves; be steadfast and self-reliant, give place to no man in that work for which you are fitted; but assert yourselves boldly, and firmly, and be assured you will succeed!

Your Alma Mater will jealously guard the honor of your profession; see that you keep it pure and unsullied. She will watch your course with sympathy in all noble aspirations, and feel a pride in your successes!

---

